# Supplementary material for: Solution-Processed OLED Based on a Mixed-Ligand Europium Complex
Source: Materials (Basel). 2023 Jan 19;16(3):959. doi: 10.3390/ma16030959 (PMC9917707; doi:10.3390/ma16030959)
Supplement: Supplementary file 1 [file materials-16-00959-s001.zip › materials-2109362-supplementary.pdf]

ELECTRONIC SUPPLEMENTARY INFORMATION

Solution-processed OLED based on a mixed-ligand europium complex

Makarii I. Kozlov,[1] Kirill M. Kuznetsov,[1] Alexander S. Goloveshkin,[2] Andrei Burlakin,[3] Maria Sandzhieva,[3] Sergey V. Makarov, [3,4,5] Elena Ilina,[6] and Valentina V. Utochnikova[1,*]

1. **TGA data** 2
2. **^{1}H NMR spectroscopy** 3
3. **XRD data** 4
4. **IR spectroscopy** 5
5. **Photoluminescence properties of thin films** 6
6. **Electroluminescence properties of thin films** 8

1. TGA data

The TGA data revealed that only Eu(tta)$_3$DPPZ·EtOH contained solvent molecules, which is eliminated in the range of 120–130°C, which results in the formation of Eu(tta)$_3$DPPZ. Thermal decomposition of the rest of the complexes begins in the range of 280-290°C which occurs in two stages; the final weight loss corresponds to the formation of a mixture of Eu_2O_3 and EuOF from Eu(tta)$_3$DPPZ, which witnesses the correctness of the ascribed composition.

The presence of coordinated solvent molecules was confirmed by IR-spectroscopy. In the spectrum of Eu(tta)$_3$DPPZ·EtOH (Fig.S1b), low intensity bands at 3100-2800 cm^{-1} are observed, corresponding to the aromatic and methane C–H vibrations of DPPZ and tta$^-$, bands at 1750-1150 cm^{-1}, corresponding to C=O vibrations in tta$^-$, and bands at 850-515 cm^{-1} corresponding to the C–F vibrations, which are also present in the anionic ligand. The presence of the low intense broad band at ca. 3400 cm^{-1}, corresponding to the vibrations of the O–H bond, witnesses the presence of coordinated ethanol molecule.

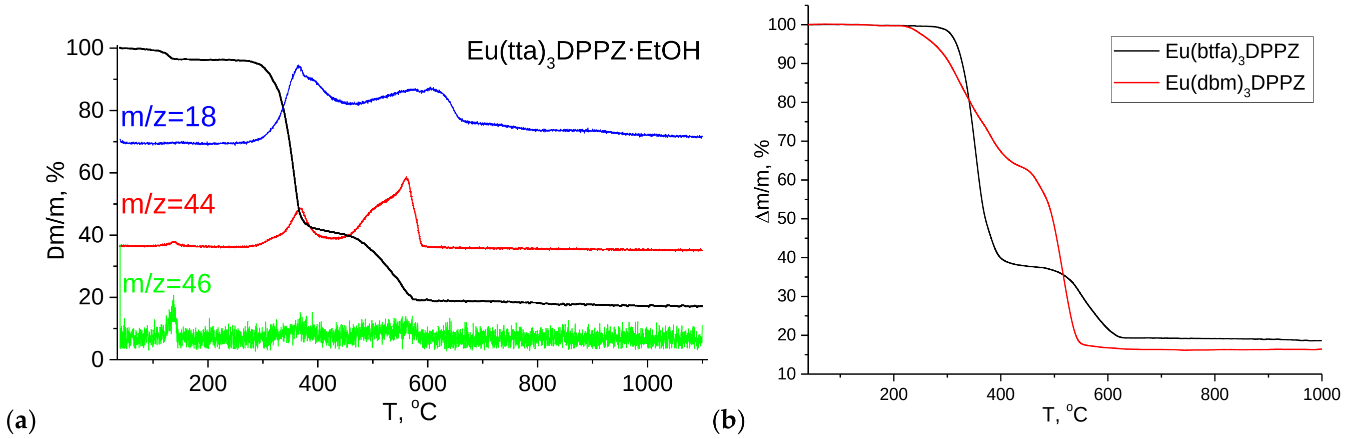

Figure S1. (**a**) TGA curve of Eu(tta)$_3$DPPZ·EtOH. Normalized ionic currents are shown in blue (m/z = 18), red (m/z = 44), and green (m/z = 46). (**b**) IR-spectrum of Eu(tta)$_3$DPPZ·EtOH. b) TGA curves of Eu(btfa)$_3$DPPZ and Eu(dbm)$_3$DPPZ

2. ^{1}H NMR spectroscopy

In order to confirm the ratio of the neutral ligand DPPZ and the anionic ligand in compound, ^{1}H NMR spectra were studied. Owing to the effect of a paramagnetic Eu^{3+} ion, broadening and a shift of proton signals are observed in the ^{1}H NMR spectra. The comparison of ^{1}H NMR spectra shows that total integrated intensity is equal to three anionic ligands per each neutral ligand. This confirms the composition of Eu(dik)$_3$DPPZ (dik = tta, btfa, dbm).

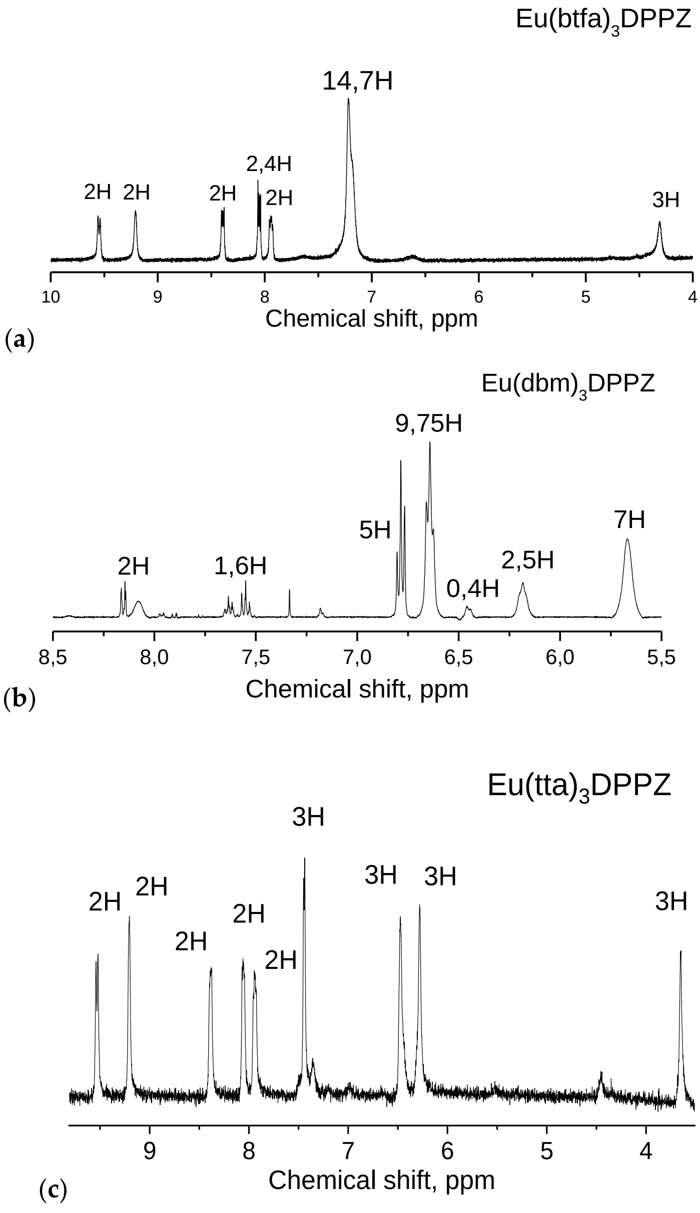

Figure S2. The ^{1}H NMR spectra of (**a**) Eu(btfa)$_3$DPPZ, (**b**) Eu(dbm)$_3$DPPZ, and (**c**) Eu(tta)$_3$DPPZ in DMSO-d^6 solution.

3. XRD data

Powder pattern of Eu(tta)$_3$DPPZ·EtOH was successfully Rietveld refined using the [Eu(tta)$_3$DPPZ]·MeCN structure (CCDC 2072867). Only the cell parameters were optimized, the atomic coordinates were taken from the structure of the single crystal and were not refined.

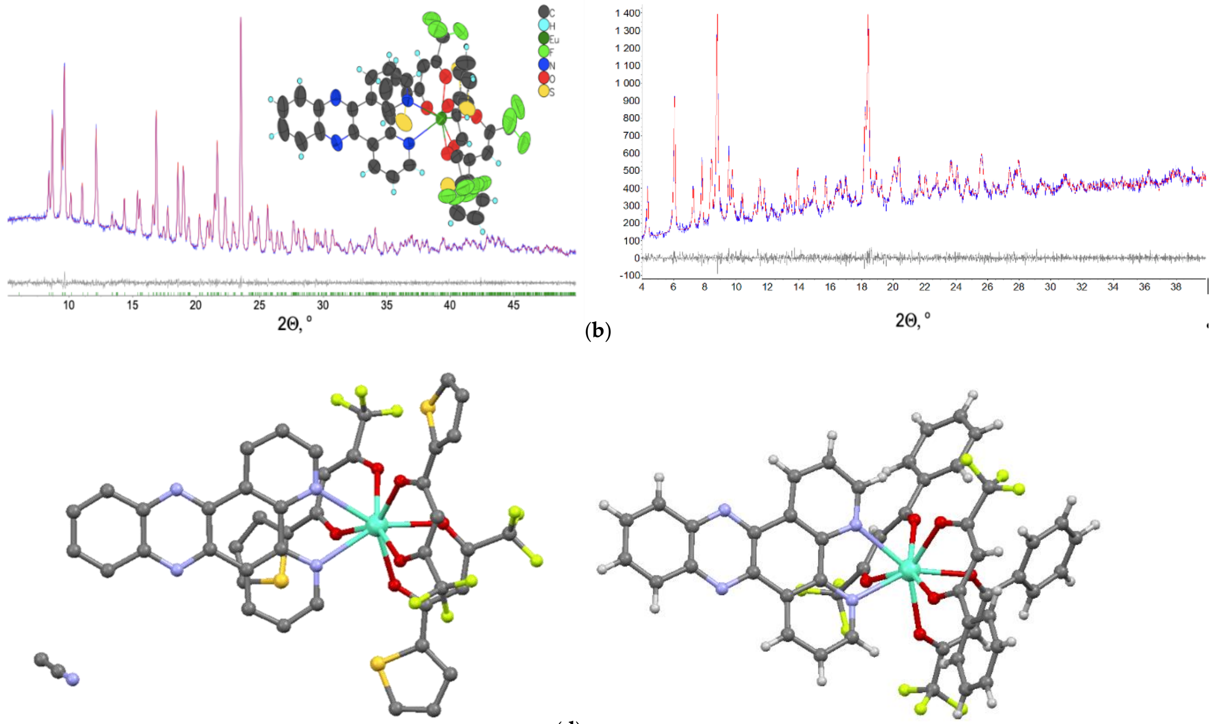

(a) (b) (c) (d)

Figure S3. (**a**) Indexed PXRD pattern of Eu(tta)$_3$DPPZ·EtOH, (**b**) Pawley fit (red line) of Eu(dbm)$_3$DPPZ (blue line) and their difference (grey curve). Molecules in the structures of (**c**) Eu(tta)$_3$DPPZ and (**d**) Eu(btfa)$_3$DPPZ.

Obtained pattern of Eu(dbm)$_3$DPPZ was successfully Pawley fitted with monoclinic cell based on Dy(dbm)$_3$DPPZ structure (CCDC 1062453). The cell parameters after refinement are following:

R-Bragg	0.175
Spacegroup	*P21/c*
Cell Volume (Å^3)	10257(9)
Lattice parameters	
a (Å)	12.289(7)
b (Å)	20.829(10)
c (Å)	40.716(19)
beta (°)	100.20(4)

4. IR spectroscopy

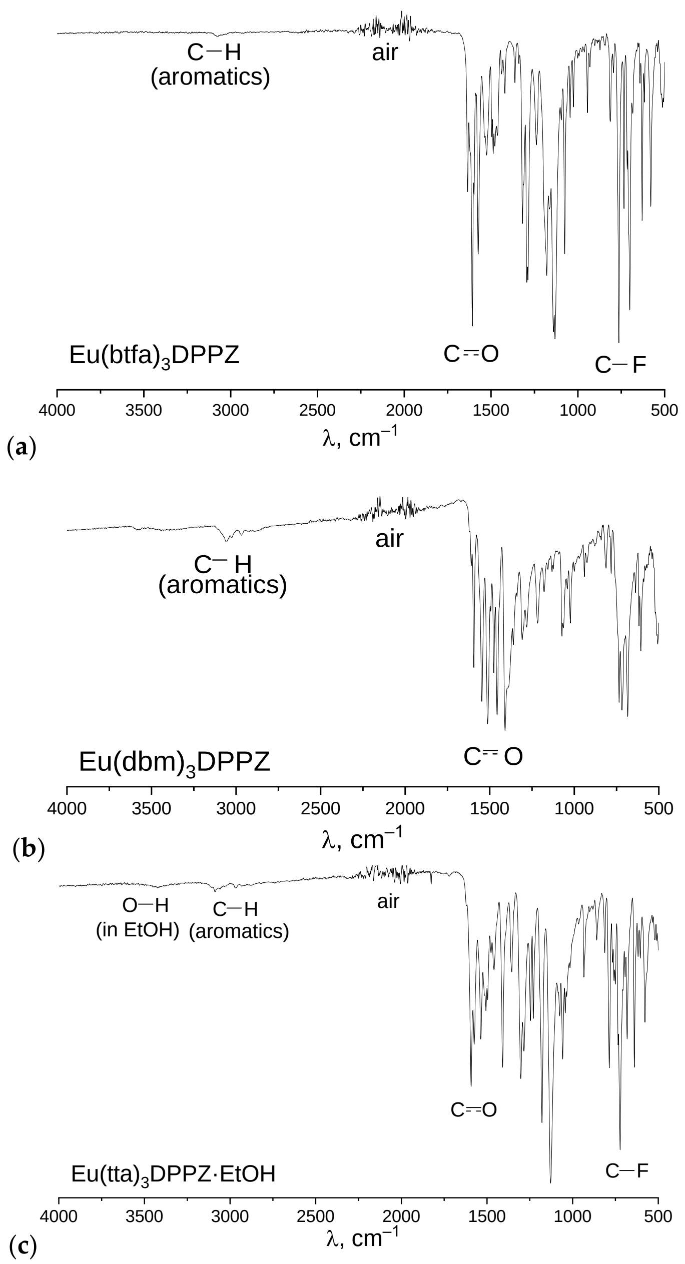

Figure S4. IR spectra of (**a**) Eu(btfa)$_3$DPPZ, (**b**) Eu(dbm)$_3$DPPZ, and (**c**) Eu(tta)$_3$DPPZ·EtOH.

5. Photoluminescence properties of thin films

We studied photoluminescent properties of pure and composite films, deposited from a 200 μL of the complex and the host solution (5 g L^{-1} concentration) at 1500 rpm for 1 min and heated at 80 °C for 20 min. After that solution-processed OLEDs were obtained with composite films used as EML.

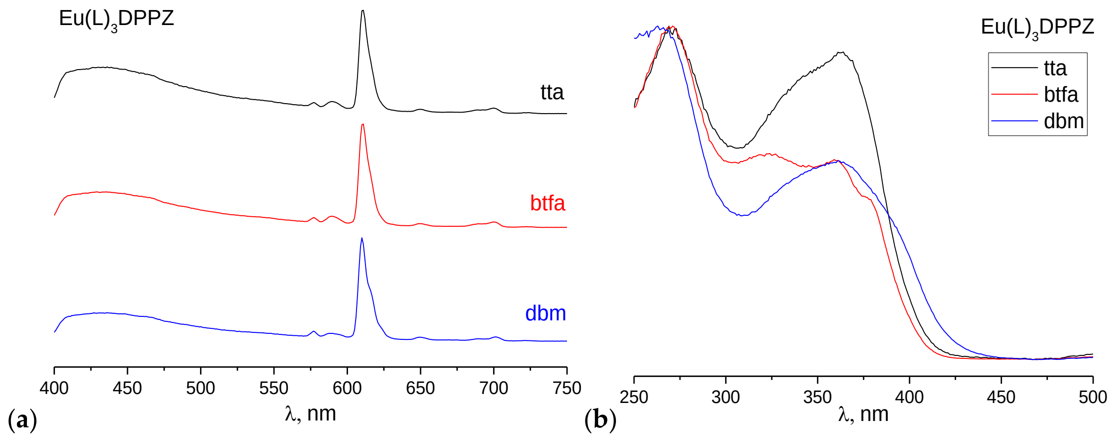

Figure S5. (**a**) Luminescence and (**b**) excitation spectra of pure thin films.

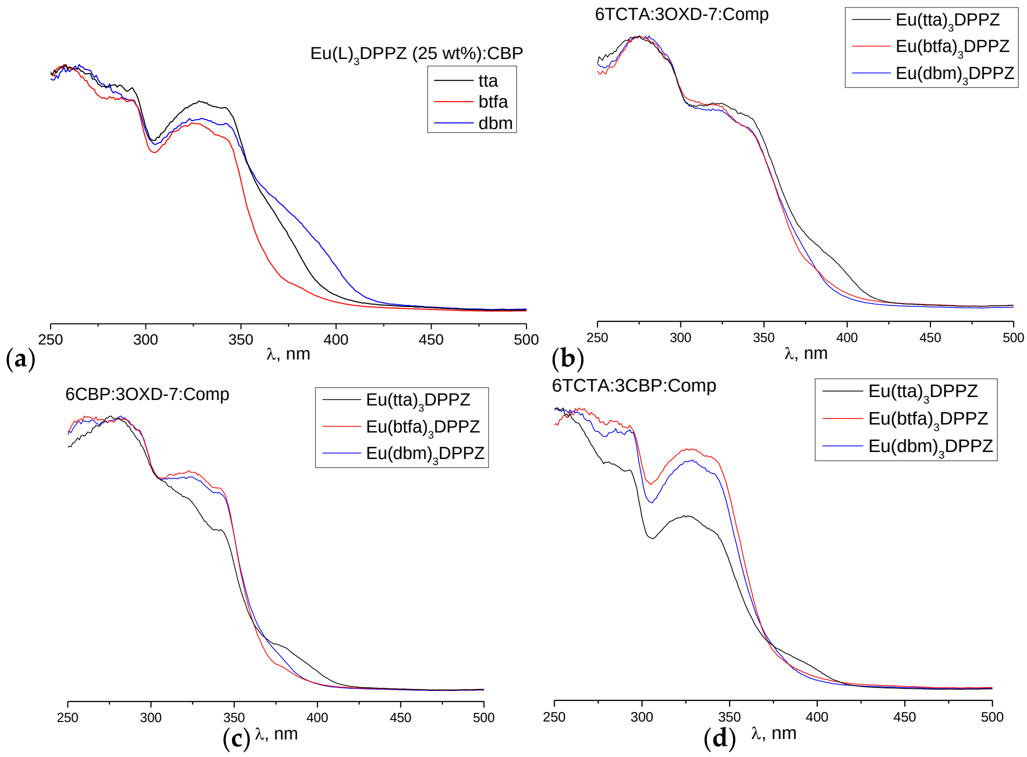

Figure S6. Excitation spectra of composite thin films of (**a**) CBP:complex = 1:3, (**b**) TCTA:OXD-7:complex = 6:3:1, (**c**) CBP:OXD-7:complex = 6:3:1, (**d**) TCTA:CBP:complex = 6:3:1.

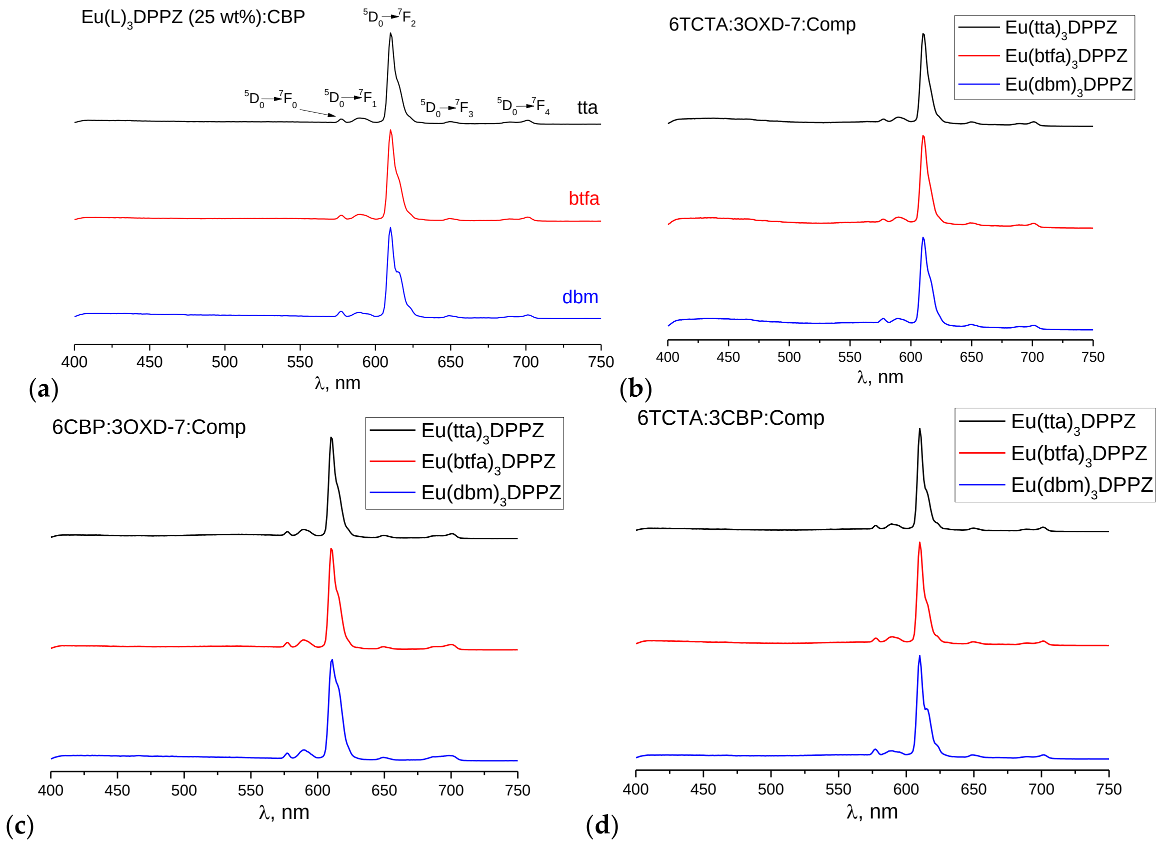

Figure S7. Luminescence spectra of composite thin films of (**a**) CBP:complex = 1:3, (**b**) TCTA:OXD-7:complex = 6:3:1, (**c**) CBP:OXD-7:complex = 6:3:1, (**d**) TCTA:CBP:complex = 6:3:1.

6. Electroluminescence properties of thin films

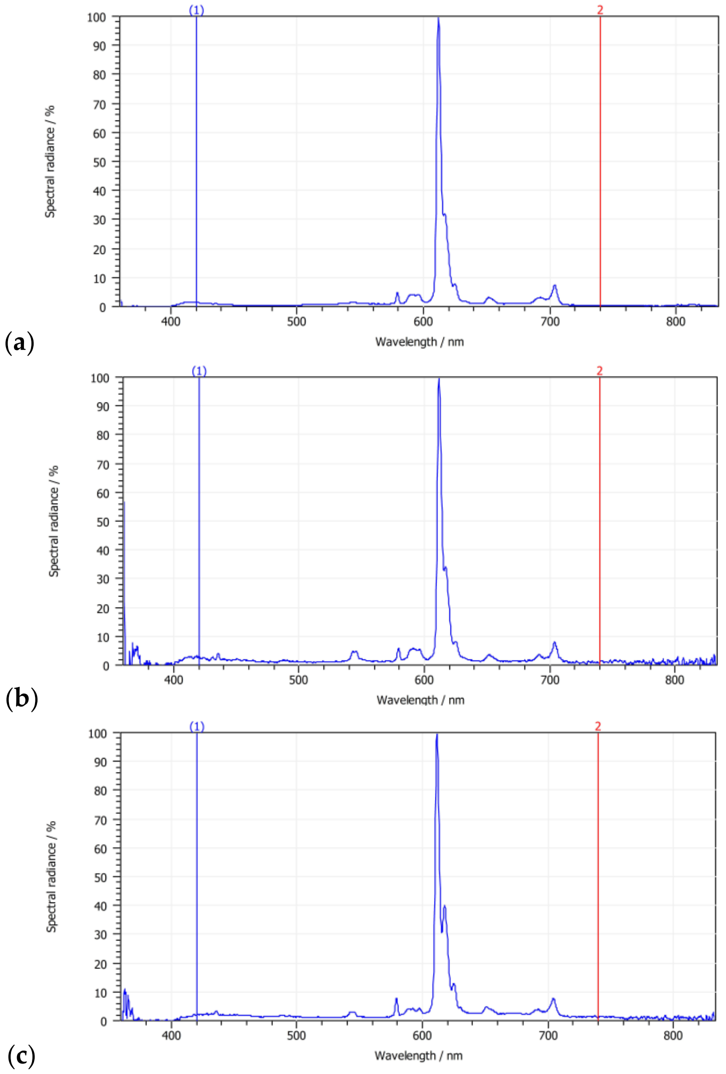

Figure S8. Electroluminescence spectra of OLEDs (**a**) S1, (**b**) S2, and (**c**) S3.
